# Toxic effect window of ovarian development in female offspring mice induced by prenatal prednisone exposure with different doses and time

**DOI:** 10.1186/s13048-023-01148-8

**Published:** 2023-04-11

**Authors:** Jing Huang, Tiancheng Wu, Yating Li, Yuanzhen Zhang, Xingjiang Yu, Dan Xu, Hui Wang

**Affiliations:** 1 Department of Obstetrics and Gynecology, Zhongnan Hospital of Wuhan University, Wuhan, 430071 China; 2 Department of Pharmacology, Wuhan University School of Basic Medical Sciences, Wuhan, 430071 China; 3 Department of Otorhinolaryngology Head and Neck Surgery, Zhongnan Hospital of Wuhan University, Wuhan, 430071 China; 4 Department of Pharmacy, School of Pharmaceutical Sciences, Zhongnan Hospital of Wuhan University, Wuhan University, Wuhan, 430071 China; 5 Hubei Provincial Key Laboratory of Developmentally Originated Disease, Wuhan, 430071 China; 6 Department of Histology and Embryology, School of Basic Medicine, Tongji Medical College, Huazhong University of Science and Technology, Wuhan, 430030 China

**Keywords:** Prednisone, Prenatal exposure, Ovarian developmental toxicity, Pre-granulosa cell, Oocyte

## Abstract

**Background:**

Prednisone is one of the most used synthetic glucocorticoids during pregnancy. Epidemiological investigations suggested that prenatal prednisone therapy could affect fetal development, but systematic studies on its effects on ovarian development and the “toxic effect window” remained scarce.

**Methods:**

In this study, by simulating clinical application characteristics, Kunming mice were given prednisone by oral gavage with different doses (0.25 or 1.0 mg/kg·d) or at different time gestational days (GD) (GD0-9, GD10-18, or GD0-18). Blood and ovaries of fetal mice were collected on GD18, and the serum estradiol level and the related function indexes of ovarian granulosa cells and oocytes were detected.

**Results:**

Compared with the control group, prenatal prednisone exposure (PPE) induced pathological injury and enhanced cell proliferation in fetal mice ovary. Furthermore, the expression of steroid synthesis functional genes in pre-granulosa cells, the oocyte function markers, and developmentally related genes was enhanced with different doses or at different time of PPE. The Hippo signaling was activated in the fetal ovary of PPE groups. The above changes were most significant in the low or high-dose and full-term PPE groups.

**Conclusion:**

PPE caused various cell developmental toxicity in the fetal ovary, especially in the low or high-dose, full-term exposure groups. The potential mechanism might be related to the activation of the Hippo signaling pathway.

**Supplementary Information:**

The online version contains supplementary material available at 10.1186/s13048-023-01148-8.

## Introduction

Since the British scholar Barker proposed that adult diseases have fetal origins in the 1990s, fetal-originated diseases have become a hot international research issue, especially the internal relationship between the environment during pregnancy and offspring development [1]. Epidemiological investigation and animal experiments have shown that adverse environment (such as exogenous toxicants/drugs, poor living habits, etc.) exposure during pregnancy can affect not only the parents’ health but also the reproductive health of the offspring during adulthood [2, 3]. During pregnancy, the mother could not avoid using drugs due to her own or fetal diseases. According to World Health Organization (WHO) statistics, in 2014, 86% of pregnant women worldwide received drug treatment, and 90% of them used more than one prescription drug. However, drug use during pregnancy could change the trajectory of fetal development through hormone imbalance, oxidative stress, and abnormal epigenetic modification and affect the development and function of multiple organs or systems of offspring [4], including the reproductive system of female offspring.

Prednisone is a synthetic corticosteroid widely used to prevent and treat various maternal diseases during pregnancy, such as autoimmune diseases, asthma, and the rejection of solid organ transplantation [5, 6]. Prednisone is the most commonly used oral glucocorticoid during pregnancy [7, 8], with a use rate of 1.3% during pregnancy [7] and a use rate of 20-60% in pregnant women with autoimmune diseases [9–11]. However, clinical studies suggested that the use of prednisone during pregnancy could increase the incidence of fetal distress, placental insufficiency [12], preterm birth [13], intrauterine growth retardation [14], significantly increased the risk of fetal oral cleft [15] and led to high cortisol levels in childhood [16]. Animal studies also reported that excessive exposure to synthetic and natural corticosteroids during pregnancy could alter the normal organ development of the fetus, including the development of the heart, brain, and kidney [17]. They might lead to the predisposition of the offspring to adult diseases such as hypertension [18]. However, there are few reports on the developmental toxicity of prenatal prednisone exposure (PPE), especially on the effect on fetal ovarian development.

Since 11β-hydroxysteroid dehydrogenase type 2 (11β-HSD2) in the placenta converts prednisone into inactive metabolites, only about 10% of the drug will cross the placenta [19]. Clinical studies have also reported an 8-10-fold decrease in the ratio of fetal prednisolone to maternal prednisolone concentrations after maternal intravenous administration [20]. Therefore, prednisone is considered safe during pregnancy, mainly for treating maternal illnesses, and can be used at any time. However, when taken at high doses or for a long time, prednisolone has been found to saturate 11β-HSD2, after which a large amount of the drug would cross the placental barrier [21]. Many clinicians suggested reducing the dose to 7.5 mg/d and avoiding doses higher than 20 mg/d in long-term treatment [15]. There are no rigorous dose-response data to suggest the optimal timing or dose of prednisone treatment during pregnancy. Therefore, the safe dose of prednisone in pregnancy is unclear.

In this study, a mice model of prenatal prednisone exposed with different doses (0.25 or 1.0 mg/kg·d) or at different gestational day (GD) (GD0-18, GD0-9, or GD10-18) were constructed according to clinical standard treatment protocols. From the perspective of the developmental changes of two types of functional ovarian cells, pre-granulosa cells and oocytes, we explored the characteristics and possible mechanism of ovarian developmental toxicity in offspring induced by PPE to provide an experimental basis for guiding rational drug use during pregnancy and further carrying out the prevention and treatment of ovarian developmental toxicity caused by prednisone.

## Materials and methods

### Chemicals

Prednisone (CAS #53-03-2, PN. MB1327) was obtained from MeiLunbio Co., LTD. (Dalian, Liaoning, China). Isoflurane was obtained from Baxter Healthcare Co. (Deerfield, IL, USA). The antibodies of proliferating cell nuclear antigen (PCNA, No. A0264) and mouse vasa homolog (MVH, No. A15624) were purchased from ABclonal Technology Co., Ltd. (Wuhan, Hubei, China). All the primers were synthesized by TIANYIHUIYUAN Biotechnology Co., Ltd. (Wuhan, Hubei, China). The enzyme-linked immunosorbent assay (ELISA) kit was obtained from the Beijing North Institute of Biological Technology (Beijing, China). The total RNA Trizol kit was purchased from Thermo Fisher Scientific Inc. (Waltham, MA, USA). Reverse transcription and quantitative real-time polymerase chain reaction (RT-qPCR) kits (Q223) were purchased from Takara Biotechnology Co., Ltd. (Dalian, Liaoning, China). Other chemicals and reagents were of analytical grade.

### Animals and treatment

Specific pathogen-free Kunming mice [2020-0018, Certification No. 42000600043615, License No. SCXK 2020-0018 (Hubei)] weighing 35 ± 12 g (females) and 25 ± 17 g (males) were obtained from the Experimental Center of the Hubei Medical Scientific Academy (Wuhan, Hubei, China). Animal experiments were performed in the Center for Animal Experiment of Wuhan University (Wuhan, Hubei, China), which the Association for Assessment and Accreditation of Laboratory Animal Care International accredits. The Committee on the Ethics of Animal Experiments of the Wuhan University School of Medicine approved the protocol (Permit No. ZN2021222). All experimental animal procedures were performed following the Guidelines for the Care and Use of Laboratory Animals of the Chinese Animal Welfare Committee.

**Fig. 1:**
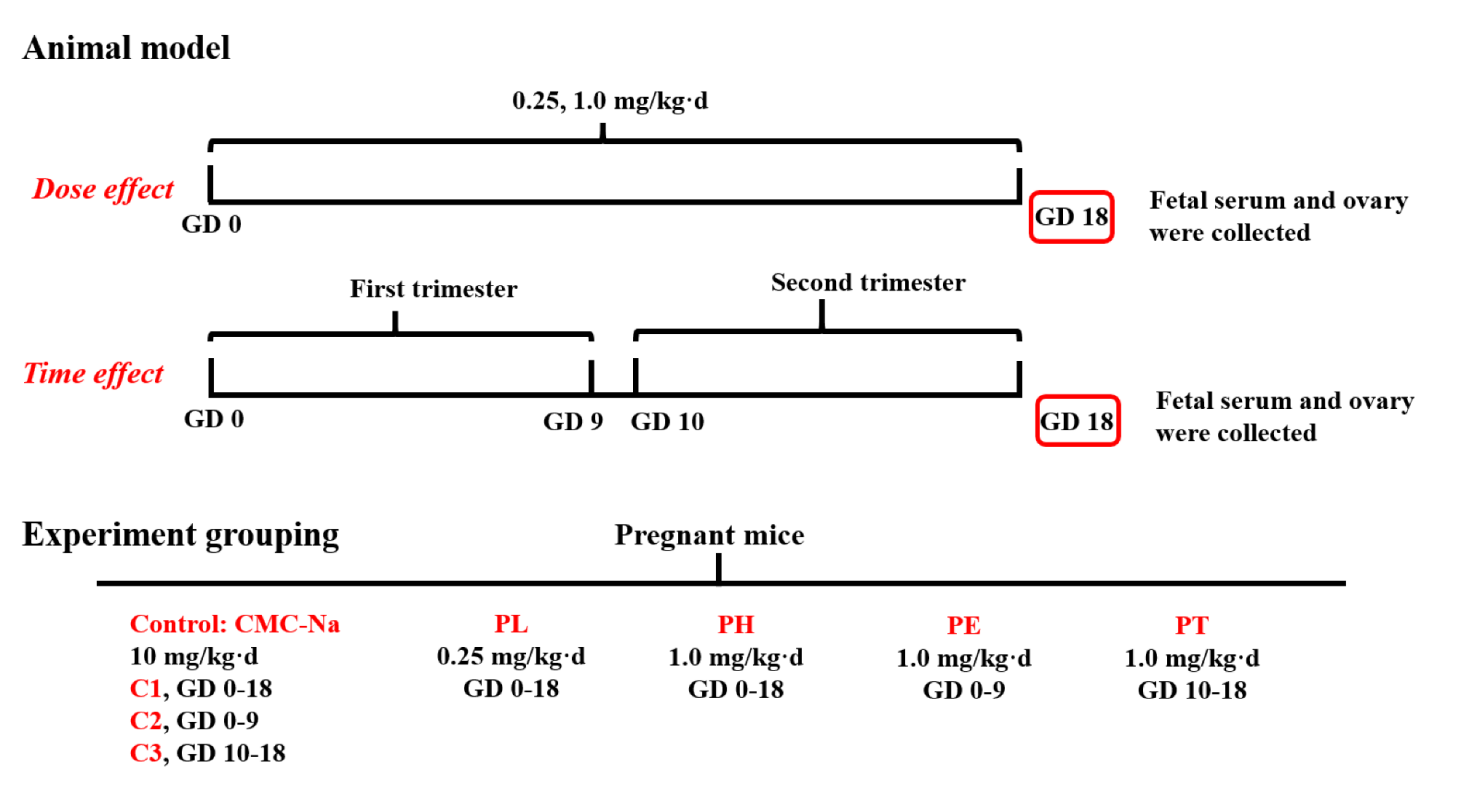
The schematic illustration of animal treatment and experiment grouping. GD, gestational day; PL, prednisone low-dose exposure; PH, prednisone high-dose exposure; PE, prednisone early high-dose exposure. PT, prednisone terminal high-dose exposure

Animals were housed (room temperature: 20–24 °C; humidity: 50-65%), acclimated, and mated. The appearance of sperm in vaginal smears or the vaginal plug confirmed mating, and the day of mating was taken as GD0. The pregnant mice were transferred to individual cages and then, as shown in Fig. 1, randomly divided into seven groups (*n* = 12) and administered prednisone with different doses (0.25 or 1.0 mg/kg·d) at GD0-18 or at different time (GD0-9 or GD10-18) with 1.0 mg/kg·d. The control group was administered the same volume of sodium carboxymethylcellulose (CMC) (10 ml/kg·d), including C1 (administered from GD0 to GD18), C2 (administered from GD0 to GD9), and C3 (administered from GD10 to GD18). On GD18, the pregnant mice were anesthetized with 3% isoflurane and then sacrificed. Pregnant mice with litter sizes of 8 to 14 were considered qualified. Each fetoplacental unit was quickly removed from the uterus, and the fetuses were weighed after being dried on filter papers. The female fetal mice were retained from each litter for subsequent analysis. The blood of female fetal mice per litter was collected, combined, and centrifuged for reserve. For the morphological purpose, one fetus per litter was randomly selected. The right ovary (one per litter, *n =* 5) was fixed in freshly prepared Bouin’s solution for 24 h before being dehydrated in alcohol and embedded in paraffin. The remaining ovaries were frozen in liquid nitrogen and stored at − 80℃ for further analysis.

### Hormonal level measurements

Serum estradiol level and fetal ovarian endogenous estrogen were measured by ELISA assay kit (Beijing North Institute of Biological Technology, Beijing, China) following the manufacturer’s protocol. The serum of female fetuses or the tissue of fetal ovary (from 3 to 4 litters) was combined as one sample.

### Hematoxylin-eosin (HE) staining

The 5-µm thickness paraffin histological sections were prepared and routinely performed with HE staining. Every 5th section of the series was saved, observed, and photographed with an Olympus AH-2 light microscope (Olympus, Tokyo, Japan), and another experimenter blindly conducted the examination and evaluation. The number of oocytes per unit square of interstitial tissue areas (10^4^ µm^2^) was calculated by examining five randomly selected sites per section (*n =* 5). Finally, we photomicrograph and evaluated relevant examination indexes (such as the maximum diameter of fetal ovaries) from each section (*n =* 5).

### Immunofluorescence (IF) analysis

The paraffin-embedded ovary tissue was cut into 5-µm sections, deparaffinized in xylene, and rehydrated. The samples were repaired in a citrate buffer (10 mM, pH 6.0) for 5 h at 65 °C and rinsed with PBS 3 times. After blocking in 5% BSA for 30 min at room temperature. One section of each ovary was chosen for subsequent analysis. For IF analysis, the sections were incubated with anti-PCNA and anti-MVH antibodies (1:100 dilution) overnight at 4 °C. Then, the sections were washed and incubated with the corresponding fluorescent secondary antibody (1:400 dilution) for 1 h in the dark room and then were stained with 4’,6-diamidino-2-phenylindole (DAPI) (1 µg/ml) for 10 min. All images were captured using an Olympus AH-2 Light Microscope (Olympus, Tokyo, Japan). Analysis of the stained images was performed using Olympus software.

### Total RNA extraction, reverse transcription, and RT-qPCR for ovary and hypothalamus

The total RNA was extracted from the ovaries using the TRIZOL reagent following the manufacturer’s protocol. The tissues of each littermate were pooled for homogenization as one sample. The concentration and purity of the total RNA were determined using a spectrophotometer (NanoDrop 2000), and the total RNA concentration was adjusted to 1 mg/ml. Single-strand cDNA was prepared from 1 mg of total RNA according to the kit’s protocol and was stored at −20℃ until use. All the primers were designed using Primer Premier 5.0 (PREMIER Biosoft International, CA). The sequences of each designed primer were queried using the NCBI BLAST database for homology comparison and are listed in Table S1. The RT-qPCR was performed using the ABI. Step One RT-PCR thermal cycler (A.B.I. Stepone, U.S.A.) in a 10-µL reaction mixture. The mRNA level of the housekeeping-gene glyceraldehyde 3-phosphate dehydrogenase (*GAPDH*) was measured and used as a quantitative control to quantify the gene transcripts more precisely. Each sample was normalized against *GAPDH* mRNA level.

### Statistical analysis

SPSS 19 (SPSS Science Inc., Chicago, Illinois), Excel (Microsoft, Redmond, WA, USA), and Prism 7.0 (GraphPad Software, La Jolla, CA, USA) were used to perform data analysis. Quantitative data were expressed as the mean ± S.E.M. the Shapiro-Wilk test was employed to examine the normality of data and the Levene test was used to evaluate the homogeneity of variances. One-way ANOVA was performed to evaluate the data of different doses, Student’s two-tailed t-test was performed to evaluate the data of different time of PPE. Statistical significance was designated at *P <* 0.05.

## Results

### Effects of different doses and time of PPE on fetal ovarian morphology and ovarian reserve

**Fig. 2:**
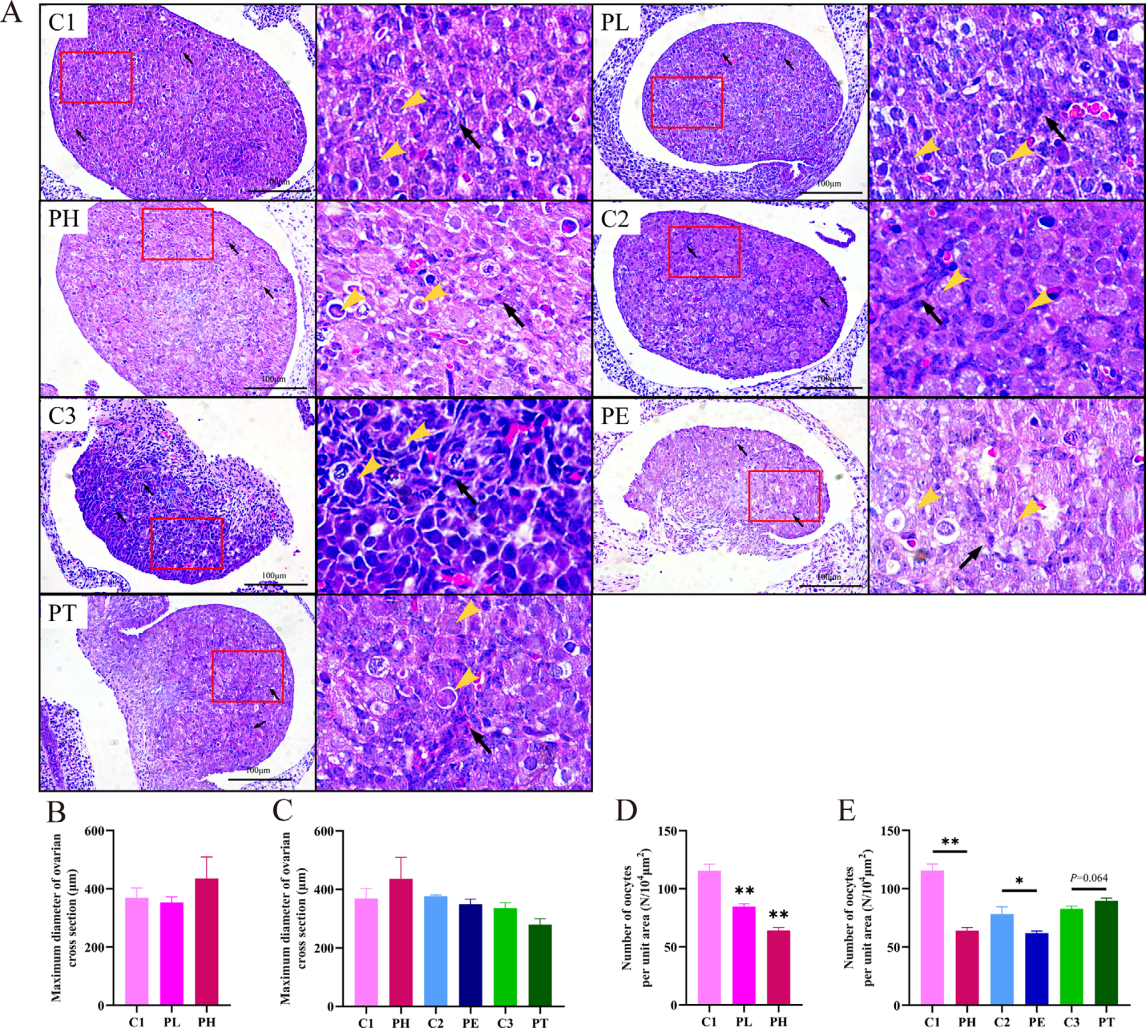
Changes in fetal ovarian morphology are caused by prenatal prednisone exposure (PPE) with different doses or at different time. (**A**) Ovarian morphology (HE, 200 ×, 400 ×, *n =* 5, yellow arrowhead showed the oocytes, black arrow showed the ovarian pre-granulosa cells); (**B, C**) Maximum diameter of ovarian cross section (µm); (**D, E**) Number of oocytes per unit area (N/10^4^ µm^2^). Mean ± S.E.M., *n =* 5. ^*^P < 0.05, ^**^P < 0.01 vs. control. C1, control of PH; PL, prednisone low-dose exposure; PH, prednisone high-dose exposure; C2, control of PE; C3, control of PT; PE, prednisone early high-dose exposure; PT, prednisone terminal high-dose exposure

The intrauterine period is a critical period for ovarian morphogenesis and functional differentiation. Firstly, we observed the effects of different doses and time of PPE on fetal ovarian morphology. The results showed that premature insertion of pre-granulosa cells into the oocyte cyst was observed in all doses of PPE groups, compared with the control group, resulting in pathological changes such as premature cleavage of the oocyte cyst and a significant decrease in the number of oocytes (Fig. 2A). In contrast, no difference between the groups was observed in the maximum cross-sectional diameter of the ovaries (Fig. 2B, C). At the same time, PPE induced a dose-dependent decrease in oocyte number (*P* < 0.01, Fig. 2D). For the time effect, compared with the control group, the fetal ovarian oocytes decreased significantly in the full-term and early exposure groups (PH, PE) (*P* < 0.01, *P* < 0.05, Fig. 2E). However, there was no significant change in the mid-late exposure group (PT) (Fig. 2E). It was suggested that PPE could cause abnormal morphological development of fetal ovaries and decrease the number of oocytes in the full-term and early exposure groups.

### Effects of different doses and time of PPE on cell proliferation and apoptosis of fetal ovary

**Fig. 3:**
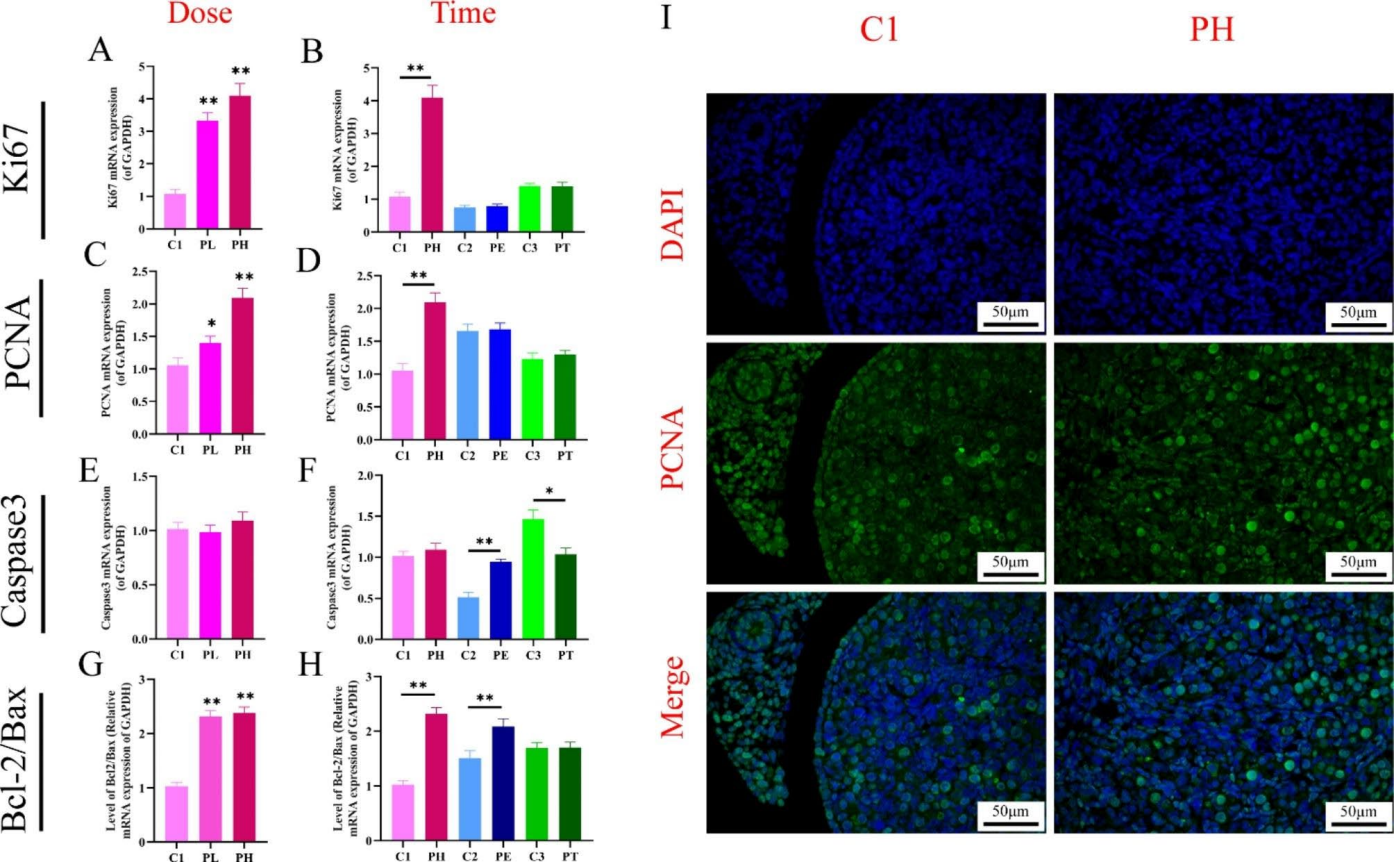
Changes of proliferation and apoptosis in fetal ovary induced by prenatal prednisone exposure (PPE) with different doses or at different time. (**A-F**) mRNA expression of Ki67, PCNA and Caspase3, *n =* 12; (**G, H**) Level of Bcl2/Bax, *n =* 12; (**I**) Protein expression of PCNA by IF (the PCNA positive cells stained by green, 400 ×, *n =* 5). Mean ± S.E.M., ^*^P < 0.05, ^**^P < 0.01 vs. control. GAPDH, glyceraldehyde phosphate dehydrogenase; PCNA, proliferating cell nuclear antigen; C1, control of PH; PL, prednisone low-dose exposure; PH, prednisone high-dose exposure; C2, control of PE; PE, prednisone early high-dose exposure; C3, control of PT; PT, prednisone terminal high-dose exposure; IF, immunofluorescence

Cell proliferation and apoptosis are critical indicators for evaluating the developmental toxicity of the organism. To determine the effects of different doses and time of PPE on cell proliferation and apoptosis of fetal ovarian, the proliferation-related genes, including Ki67, PCNA, and apoptosis-related genes Caspase3, Bcl-2, Bax were selected as the evaluation indexes. RT-qPCR results showed that for the dose-effect, compared with the control group, PPE can dose-dependently (PL, PH) promote mRNA expression of Ki67 and PCNA in the fetal ovary (*P* < 0.05, *P* < 0.01, Fig. 3A, C) and increased the ratio of Bcl-2/Bax (*P* < 0.01, Fig. 3G), while no significant difference was found in the mRNA expression of Caspase3 (Fig. 3E). For the time effect, the ratio of Bcl-2/Bax was significantly increased in both the full-term pregnancy group (PH) and the early pregnancy group (PE) (*P* < 0.01, Fig. 3H), the Ki67 and PCNA mRNA levels in PH group were promoted at the same time (*P* < 0.01, Fig. 3B, D), while the Caspase 3 mRNA level was decreased in the PT group and promoted in the PE group (*P* < 0.05, *P* < 0.01, Fig. 3F). Further, PCNA immunofluorescence staining of C1 and the full-term pregnancy group (PH) revealed that the protein expression trend was consistent with the gene expression (Fig. 3I). It was suggested that PPE could promote cell proliferation and inhibit apoptosis of fetal ovarian to a certain extent. These effects were evident at low and high doses and in the full-term exposure groups.

### Effects of different doses and time of PPE on steroid synthesis function in fetal ovaries

**Fig. 4:**
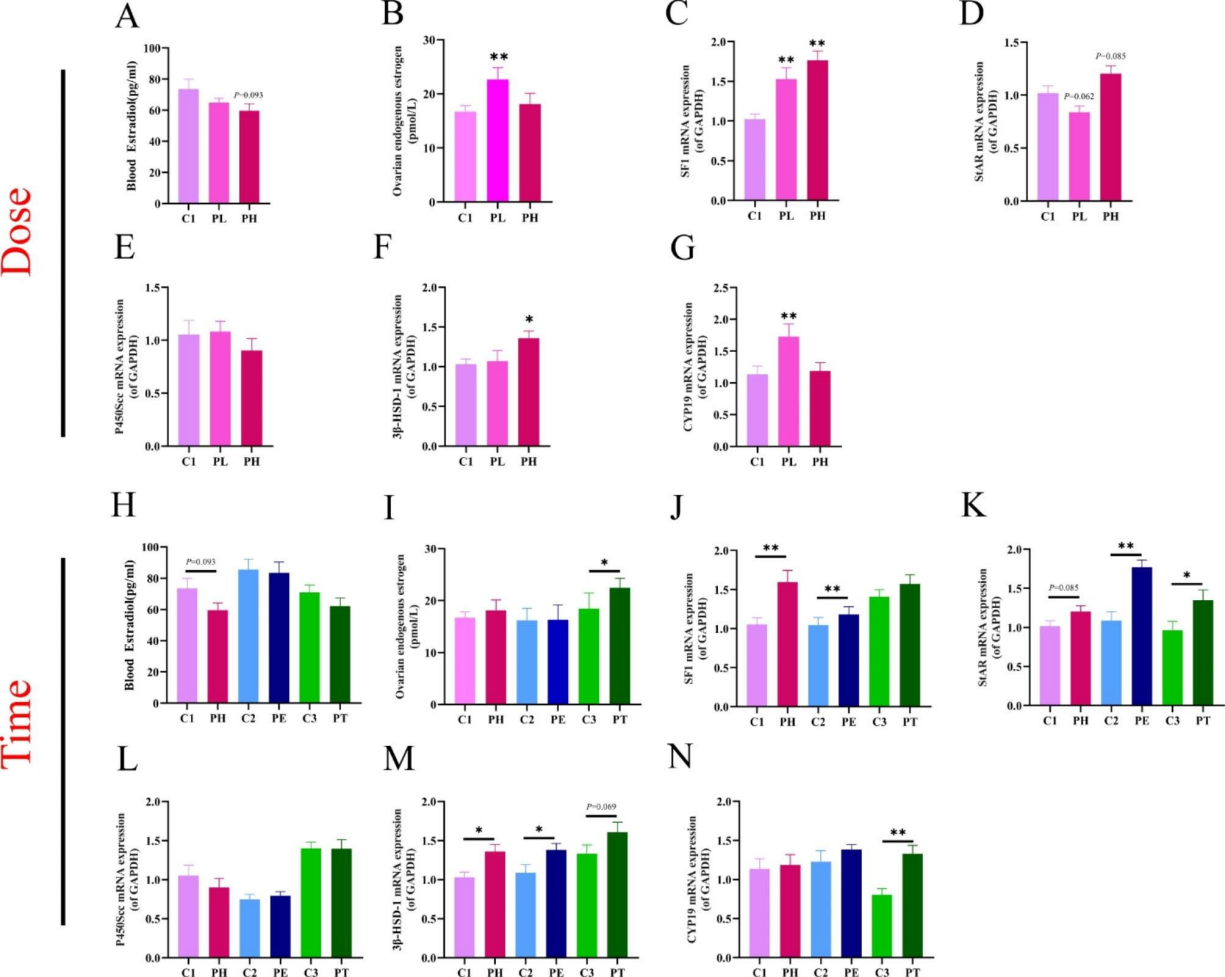
Changes of estradiol synthesis function in fetal ovary induced by prenatal prednisone exposure (PPE) with different doses or at different time. (**A, H**) Blood estradiol levels by ELISA; (**B, I**) Ovarian endogenous estradiol levels by ELISA (*n* = 6); (**C-G, J-N**) mRNA expression of SF1, StAR, P450scc, 3β-HSD1, and CYP19 at different doses/times (*n =* 12). Mean ± S.E.M., ^*^P < 0.05, ^**^P < 0.01 vs. control. GAPDH, glyceraldehyde phosphate dehydrogenase; SF1, splicing factor 1; StAR, steroidogenic acute regulatory protein; P450scc, cytochrome P450 cholesterol side chain cleavage; 3β-HSD1, 3β-hydroxysteroid dehydrogenase-1; CYP19, cytochrome P450 family 19; C1, control of PH; PL, prednisone low-dose exposure; PH, prednisone high-dose exposure; C2, control of PE; PE, prednisone early high-dose exposure; C3, control of PT; PT, prednisone terminal high-dose exposure

Estradiol is an essential class of sex hormones synthesized and secreted by ovarian pre-granulosa cells. We observed PPE’s effects on fetal blood levels and endogenous ovarian estradiol, and the mRNA expression of the ovarian steroid synthase system. ELISA results showed that fetal blood estradiol levels were not significantly changed in any PPE groups (Fig. 4A, H). However, fetal ovarian endogenous estrogen levels were significantly increased in the PPE full-term, low-dose group (PL) and mid-late pregnancy group (PT) (*P* < 0.01, *P* < 0.05, Fig. 4B, I). Further detection of ovarian steroid synthase revealed that for the dose effect, the mRNA expression of SF1 and 3β-HSD1 in the PH group and SF1 and CYP19 in the PL group were significantly higher compared with the control group (*P* < 0.01, *P* < 0.05, Fig. 4C-G). For the time effect, some steroid synthase mRNA levels were increased in all PPE groups (*P* < 0.01, *P* < 0.05, Fig. 4J-N), but CYP19 was only increased in PT group (*P* < 0.01, Fig. 4N). In conclusion, PPE can promote the function of estradiol synthesis in fetal mice to some extent. The effect was most obvious in low-dose, full-term, and mid-late pregnancy groups.

### Effects of different doses and time of PPE on fetal ovarian oocytes and follicle development

**Fig. 5:**
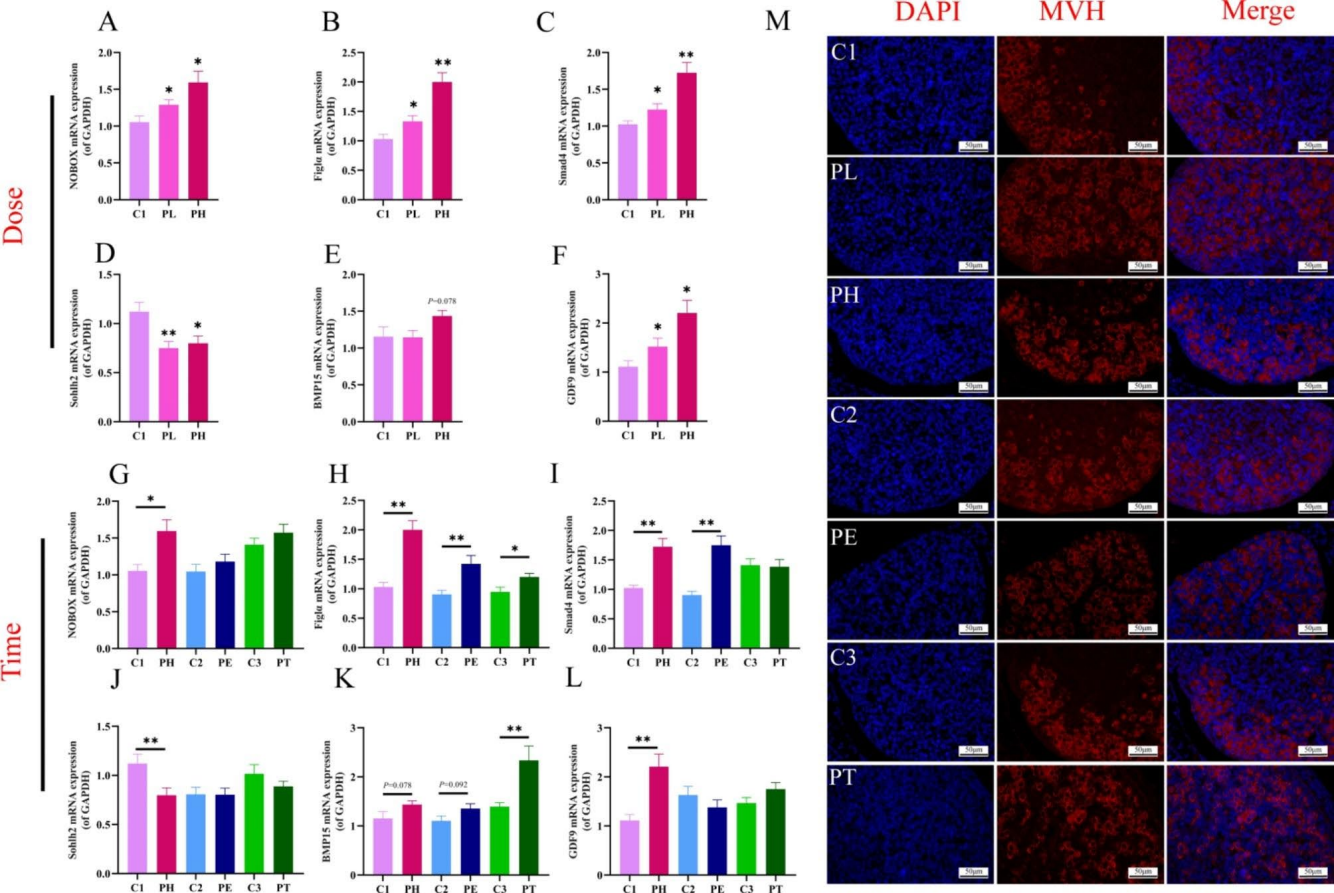
Changes of oocyte function in fetal ovary induced by prenatal prednisone exposure (PPE) with different doses or at different time. (**A-L**) mRNA expression of NOBOX, Figlα, Smad4, Sohlh2, BMP15, and GDF9; (**M**) Protein expression of MVH by IF (the MVH positive cells were stained by red, 400 ×, *n* = 5). Mean ± S.E.M., *n =* 12. ^*^P < 0.05, ^**^P < 0.01 vs. control. GAPDH, glyceraldehyde phosphate dehydrogenase; NOBOX, endogenous retroviral envelope genes; Figlα, factor in the germ line; Smad4, SMAD family member 4; Sohlh2, Spermatogenesis and oogenesis basic helix-loop-helix transcription factor 2; BMP15, bone morphogenetic protein 15; GDF9, growth and differentiation factor 9; C1, control of PH; PL, prednisone low-dose exposure; PH, prednisone high-dose exposure; C2, control of PE; PE, prednisone early high-dose exposure; C3, control of PT; PT, prednisone terminal high-dose exposure; MVH, mouse vasa homolog; DAPI, 4’,6-diamidino-2-phenylindole

The normal development of oocytes is essential to ensure the normal reproductive function of the ovary. We further examined the effects of different doses and time of PPE on the mRNA expression of markers related to fetal ovarian oocyte development, including NOBOX, Smad4, Figlα, and Sohlh2. The mRNA expression of NOBOX, Figlα, and Smad4 was significantly increased in PL and PH groups (*P* < 0.05, *P* < 0.01, Fig. 5A-C), while that of Sohlh2 was decreased significantly in all dosage groups (*P <* 0.05, *P* < 0.01, Fig. 5D). For the time effect, the mRNA expression of NOBOX, Figlα, and Smad4 in the PH group was higher than that of the PE and PT groups, while the expression of Sohlh2 was reduced significantly (*P* < 0.05, *P* < 0.01, Fig. 5G-J). To further confirm the effect of PPE on oocyte development, we selected a stable marker of MVH for germ cells undergoing immunofluorescence staining. The results showed that the expression of MVH protein in oocytes was significantly elevated in the full-term, low, or high-dose PPE groups (Fig. 5M). In conclusion, PPE may significantly promote oocyte development at low-dose, high-dose, and full-term exposure groups.

Oocytes can promote follicle assembly and maturation by expressing different regulatory factors. Therefore, we also observed the effect of PPE on the mRNA expression of GDF9 and BMP15 (the critical factors for fetal follicle assembly and development). For the dose effect, compared with the control group, the mRNA expression of GDF9 was significantly increased in PL and PH groups (*P* < 0.05, Fig. 5F), but there was no significant change in BMP15 (Fig. 5E). For the time effect, the mRNA expressions of GDF9 in PH and BMP15 in PT were significantly increased (*P* < 0.01, Fig. 5K, L). In conclusion, PPE promoted the expression of development genes associated with fetal ovarian follicles and was most significant at high dose, mid-late, or full-term exposure groups.

### Effects of different doses and time of PPE on the fetal ovarian Hippo signaling pathway

**Fig. 6:**
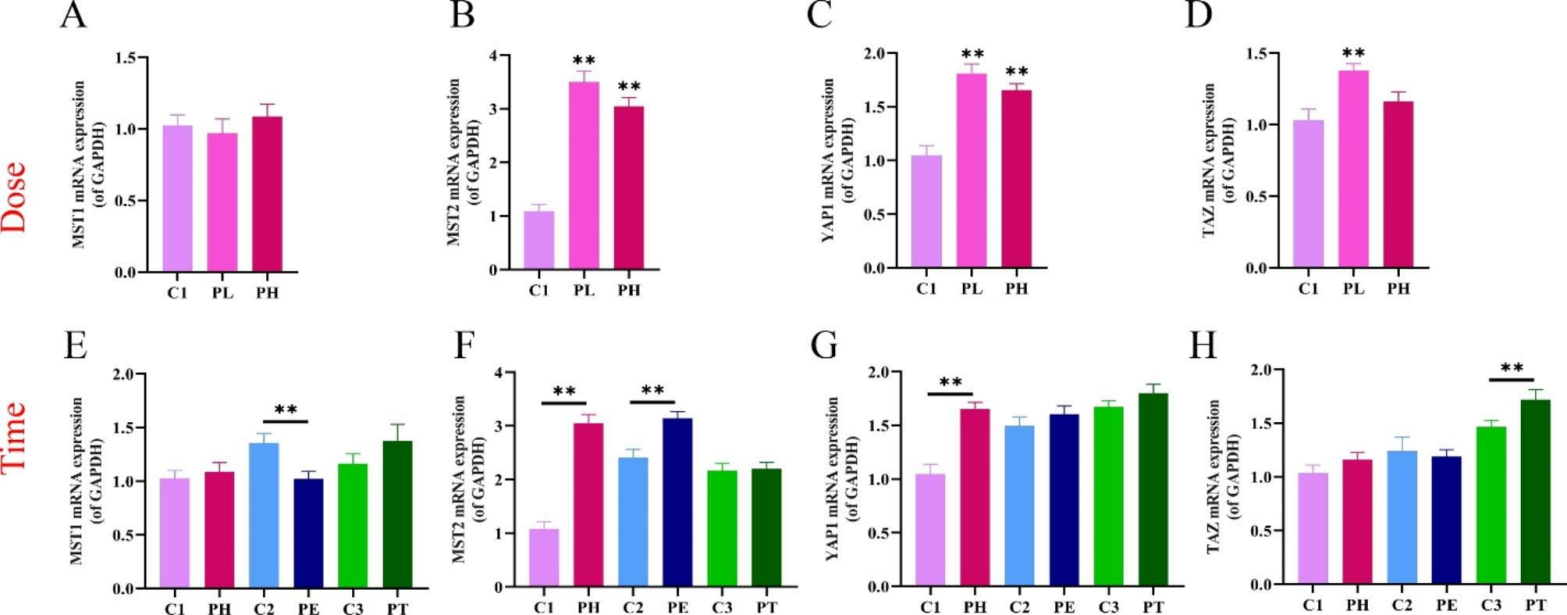
Changes of the Hippo signaling pathway in fetal ovary induced by prenatal prednisone exposure (PPE) with different doses or at different time. (**A-H**) mRNA expression of MST1, MST2, YAP1and TAZ. Mean ± S.E.M., *n =* 12. ^**^P < 0.01 vs. control. GAPDH, glyceraldehyde phosphate dehydrogenase; MST1, multiple tumor suppressor 1; MST2, multiple tumor suppressor 2; YAP1, yes-associated protein 1; TAZ, transcriptional co-Activator with PDZ-binding motif; C1, control of PH; PL, prednisone low-dose exposure; PH, prednisone high-dose exposure; C2, control of PE; PE, prednisone early high-dose exposure; C3, control of PT; PT, prednisone terminal high-dose exposure

The ovarian Hippo signaling pathway and its effector YAP1 are essential for the functional development of ovarian cell growth and differentiation, follicle activation, and steroid synthesis [22]. To investigate the possible mechanism of the effect of PPE on ovarian development and function in the offspring, we examined the indicators related to the Hippo signaling pathway that plays a crucial role in ovarian development during embryonic development. The results showed that compared with the control group, MST1 was not altered for the dose-effect, and the mRNA expression of MST2, YAP1, and TAZ in the PL group, and MST2 and YAP1 in the PH group were significantly increased (*P* < 0.01, Fig. 6A-D). For the time effect, the mRNA expression of MST2 and YAP1 was elevated more significantly in the full-term exposure group (PH) than in the early and mid-late exposure groups (PE and PT) (*P* < 0.01, Fig. 6E-H). In conclusion, PPE could cause activation of the Hippo signaling pathway in fetal mice ovaries, and it was most significant in the low or high-dose and full-term exposure groups.

## Discussion

### Clinical correlation analysis of treatment time and dose in PPE mice in this study

Corticosteroids are clinically powerful anti-inflammatory agents used in many patients with rheumatic diseases. Among them, prednisone and prednisolone can be metabolized through the placental 11β-HSD2, and the fetus is exposed to only about 10% of the maternal dose [19, 21, 23], making prednisone the medication of choice for the treatment of immune diseases in pregnant women [24]. Clinical studies have found that maternal use of prednisone during pregnancy can range from 3 to 292 days, and the daily dose of prednisone varies widely from as little as 1 mg/d to as much as 60 mg/d, with a median dose of 7 mg/d [11, 25]. If converted according to the body weight of pregnant women (with 70 kg body weight) and the ratio of human to mouse body surface area (37:3) [26], the daily exposure to prednisone during pregnancy in the population (7/70 = 0.1 mg/kg·d) is equivalent to 1.23 mg/kg·d in mice. Therefore, for the dose-effect study, we designed two dosage groups (PL, PH) of 0.25 and 1.0 mg/kg·d to simulate the commonly used clinical doses. Fetal ovarian development has several important time nodes. In humans, primordial germ cells reach the gonadal ridge from the endoderm of the yolk sac in the seventh week of gestation [27]. In mice, they arrive at the gonadal ridge at GD10 and undergo extensive cell proliferation, apoptosis, and meiosis, gradually forming mature ovarian morphology such as pre-granulosa cells, primordial follicles, etc. [28]. Meanwhile, various cells proliferate and differentiate to ensure the normal functional development of the ovary, such as the first peak of estrogen synthesis function at approximately GD14 [29]. Therefore, we constructed early and mid-late exposure groups (PE, PT) and a full-term exposure group (PH) to observe the effects of exposure at different pregnancy periods on fetal mice’s ovarian development. In conclusion, in this study, we constructed a PPE mouse model with different doses and time by simulating the characteristics of clinical drug administration and ovarian development. We conducted studies on fetal ovarian morphological and functional developmental toxicities, which can help explore the patterns of the ovarian developmental toxicity of prednisone.

### PPE caused abnormal cell proliferation, apoptosis and morphological development of fetal ovary

The intrauterine period is a period of high cell proliferation in ovarian tissue, essential for forming normal ovarian morphology and function. Under physiological conditions, mouse oocytes stop division at ED13.5 and enter meiosis to form oocytes. These oocytes are closely linked in clusters called germ cell cysts [30, 31]. The germ cell cysts are destroyed shortly after birth and a large number of germ cells die programmed. At the same time, somatic cells proliferate and burst into the nest to enclose the oocyte, thus producing a primordial follicle [32]. This change is required for a proper association between germ cells and pre-granulosa cells to facilitate primordial follicle formation [33]. Abnormalities in the proliferation and apoptosis of fetal ovarian cells have been reported in adverse environments during pregnancy [34, 35]. PCNA is an essential cell proliferation marker expressed in both oocytes and granulosa cells, serving as an early marker of granulosa cell proliferation [36] and playing an important role in primordial follicle assembly [37]. Ki67 also plays an essential role in the toxic effects of adverse environment-induced ovarian development and is widely expressed in ovarian somatic cells [38]. Caspase3 is required to promote granulosa cell apoptosis but is relatively unimportant for regulating germ cell apoptosis [39]. Bax itself can promote cell apoptosis, and Bax can also form a heterodimer with Bcl-2, which can promote cell apoptosis by inhibiting Bcl-2 [40]. Most animal studies have shown that the expression of Bcl-2 and Bax genes plays a crucial role in ovarian germ cell death [41, 42].

In this study, we found that fetal ovarian PCNA and Ki67 expressions were only increased in the full-term exposure group, the Bcl-2/Bax ratio was up-regulated in the PH and PE groups, and Caspase3 expression was increased in the early pregnancy and decreased in the mid-late pregnancy. It was suggested that full-term PPE promoted the proliferation of germ cells and pre-granulosa cells and inhibited the apoptosis of germ cells. Exposure in mid-late pregnancy inhibited the apoptosis of pre-granulosa cells, while in early pregnancy promoted the apoptosis of pre-granulosa cells and inhibited the apoptosis of germ cells. The imbalance of proliferation and apoptosis of pre-granulosa cells and germ cells is not conducive to the formation of a primordial follicle pool. In this study, a series of pathological changes, such as premature cleavage of oocyte cysts and pre-granulosa cell bursting into cysts to wrap oocytes, were also observed in the fetal ovaries of the full-term PPE group. It is suggested that PPE can cause the imbalance of proliferation and apoptosis of fetal ovarian cells and advance their morphological development. In addition, full-term PPE has a greater influence on fetal ovaries than in early and mid-late pregnancy exposure. The fetal mouse ovaries develop from GD9.5 [28, 43], and there might be self-adaptive regulation of organ development during the embryonic period [36]. Only 10% of the active drug passes through the placenta during mid-late pregnancy administration when the cumulative dose of prednisone does not reach a level capable of saturating the placental 11β-HSD2 [19, 21]. It is suggested that this might be the reason why the effect of PPE on the fetus in early and mid-late pregnancy is less than in full-term pregnancy.

### PPE caused abnormal ovarian multicellular function in fetal mice

Specific markers and functional genes exist for different cells in the ovary. They can be used to reflect the developmental status of each cell directly. The pre-granulosa cells are located in the cortical region and are mainly involved in the synthesis and secretion of steroids [44, 45]. SF1 is an upstream transcriptional activator of several enzymes (e.g., StAR and CYP19) that regulate estrogen biosynthesis and reflect the function of estrogen synthesis [46]. Fetal circulating estrogen levels are affected by maternal estrogen levels and placental estrogen synthesis [47, 48], while fetal ovaries produce endogenous estrogen to regulate primordial follicle formation [49]. In our study, although there was no significant change in fetal blood E2, endogenous ovarian estrogen and CYP19 mRNA level were significantly increased in PL and PT groups. Other PPE exposure groups also caused elevated expression of partial steroid synthetase. It was suggested that PPE promotes estrogen synthesis in pre-granulosa cells, which is most obvious when exposed in low-dose and full-term or mid-late pregnancy. This may be because full-term PPE promotes the proliferation of pre-granulosa cells, while mid-late pregnancy exposure inhibits apoptosis of pre-granulosa cells, thus affecting the expression of CYP19 in fetal ovarian pre-granulosa cells. However, the specific mechanism needs to be further explored.

Normal germ cell development and primordial follicle formation determine the reproductive lifespan of mammalian females. The normal primordial formation highly depends on the timely and synchronized development of oocytes and follicular precursor somatic cells (i.e., pre-granulosa cells) [50]. Various genes (e.g., Figlα [51, 52], Nobox [53], etc.) and hormones (e.g., estrogen [36], etc.) are known to be involved in germ cell development for primordial follicle formation and normal ovarian development. Oocytes also promote primordial follicle growth and activation by expressing different regulatory factors (e.g., GDF9 [54, 55], BMP15 [56], etc.).

In the present study, the mRNA expressions of oocyte marker genes (Figlα, Nobox) and follicle-promoting gene GDF9 were significantly increased in all dose and full-term groups. It is suggested that PPE stimulates premature and excessive development of fetal ovarian oocytes. The balance between quiescence, death, and activation of primordial follicles is essential for maintaining an appropriate reproductive lifespan [57]. Once primordial follicles are formed in the ovary, most remain quiescent to preserve the length of the female reproductive lifespan [27, 58]. Therefore, we hypothesized that early and excessive development of fetal ovarian oocytes due to PPE might lead to excessive follicle depletion of the offspring after birth, accelerated depletion of ovarian reserve, shortened reproductive lifespan, and susceptibility to premature ovarian failure in adulthood [56]. The study is to be further developed at a later stage.

### Hippo signaling pathway might be involved in PPE-induced ovarian developmental toxicity in fetal mice

Studies have shown that the Hippo signaling pathway is critical to ovarian function by regulating ovarian cell proliferation and follicle activation and survival [59]. Key molecules of the Hippo signaling pathway, including MST1/2, YAP, and TAZ, play critical regulatory roles in ovarian granulosa cells [60–62] and can promote granulosa cell proliferation [63]. Follicular development has been associated with elevated levels of YAP1 and TAZ and suppressing levels of MST1 and L-amino acid transporters [60]. The knockdown of the YAP1 has been shown to inhibit primordial follicle activation, while its overexpression led to the opposite trend [60]. Thus, the Hippo signaling pathway regulates granulosa cell proliferation and ovarian follicle activation. In the present study, we found that MST2 and YAP1 were significantly elevated in the PH group, along with the enhanced proliferation of ovarian cells in fetal mice and enhanced oocyte activation. It is suggested that the Hippo signaling pathway might be involved in the enhanced proliferation of fetal ovarian granulosa cells and premature and excessive activation of primordial follicles due to PPE. In conclusion, PPE might promote pre-granulosa cell proliferation through activation of the Hippo signaling pathway in the fetal ovary and disrupt the balance between primordial follicle activation and quiescence, resulting in excessive depletion of the primordial follicular pool.

## Conclusion

In summary, in this study, we first constructed a mouse model of PPE with different doses or time by simulating the application characteristics of clinical prednisone to investigate the effects of maternal PPE on ovarian morphology and function in fetal mice. We found that low or high-dose, full-term PPE resulted in advanced ovarian morphological development and enhanced ovarian cell proliferation in offspring, accompanied by enhanced steroid synthesis in the pre-granulosa cells, over-activation of primordial follicles, premature and excessive oocyte development, and activated Hippo pathway. The present study provides a comprehensive observation of the developmental toxicity of fetal mice ovaries, the pattern of changes, and the possible mechanisms due to different doses or time of PPE. It gives new ideas to guide the safe use of the medicine during pregnancy and the experimental basis for further elucidating prednisone-induced ovarian developmental toxicity and its intrauterine programming mechanisms.

## Electronic supplementary material

Below is the link to the electronic supplementary material.

**Table S1**: Oligonucleotide primers and PCR conditions of the mouse in real-time quantitative PCR

## Data Availability

The analyzed data sets generated during the study are available from the corresponding author upon reasonable request.
