# Supplementary material for: Toxic effect window of ovarian development in female offspring mice induced by prenatal prednisone exposure with different doses and time
Source: J Ovarian Res. 2023 Apr 11;16:71. doi: 10.1186/s13048-023-01148-8 (PMC10088227; doi:10.1186/s13048-023-01148-8)
Supplement: Supplementary file 1 — Table S1: Oligonucleotide primers and PCR conditions of the mouse in real-time quantitative PCR [file 13048_2023_1148_MOESM1_ESM.docx]

**Table S1. Oligonucleotide primers and PCR conditions of the mouse in real-time quantitative PCR.**

| Genes | Forward primer | Reverse primer | Annealing |
| --- | --- | --- | --- |
| *GAPDH* | GCAAGTTCAATGGCACAG | GCCAGTAGACTCCACGACA | 63℃, 30 s |
| *PCNA* | CACTCCACTGTCTCCTACA | GCCTAAGATGCTTCCTCATC | 60℃, 30s |
| *Ki67* | CTGGTCTCAAAGGACCAATC | CTCTTCATCTGCCTCTACTTTC | 60℃, 30s |
| *Caspase3* | GATATTGTTAGCGGTTCCTGTG | AACCAGGCTGTCGTCTAA | 60℃, 30s |
| *Bcl2* | ATGCCTTTGTGGAACTATATGGC | ATGCCTTTGTGGAACTATATGGC | 60℃, 30s |
| *Bax* | GGAGATGAACTGGACAGCAAT | GAAGTTGCCATCAGCAAACAT | 60℃, 30s |
| *SF1* | CCAGTACGGCAAGGAAGA | GAGGCTGAAGAGGATGAGGA | 63℃, 30 s |
| *StAR* | GGGAGATGCCTGAGCAAAGC | GCTGGCGAACTCTATCTGGGT | 63℃, 30 s |
| *P450scc* | GCTGCCTGGGATGTGATTTTC | GATGTTGGCCTGGATGTTCTTG | 63℃, 30 s |
| *3β-HSD1* | TCTACTGCAGCACAGTTGAC | ATACCCTTATTTTTGAGGGC | 58℃, 30 s |
| *CYP19* | ATGGGCCTCCTTCTCCTGAT | CAGGCACTTCCAATCCCCAT | 60℃, 30 s |
| *NOBOX* | GACATGGGACCTCAGGATTA | GAGTCTTCTGGTGGTAGAAATG | 60℃, 30 s |
| *Figlα* | AGAGCGTGAGCGGATAAA | CCAGAACACAGCCAAGTATC | 62℃, 30s |
| *BMP15* | GTGCTCAGGCTAAACTTCTT | GGAGGGAACACTGGTTATTT | 60℃, 30s |
| *GDF9* | GATGTGACCTCCCTCCTTCA | GCCTGGGTACTCGTGTCATT | 60℃, 30s |
| *Smad4* | AGTAATCGCGCATCAACGGA | TGTGAACTGGCCTTGTGGAA | 62℃, 30 s |
| *Sohlh2* | TCTCAGCCACATCACAGAGG | GGGGACGCGAGTCTTATACA | 60℃, 30 s |
| *Mst1* | GGACTTATCATCCCGAACAGAG | CAGCTGCATGACCTTGTTAATC | 60℃, 30 s |
| *Mst2* | CCCACCACCAACATTCAG | CATTGTGCCCACGCTTTC | 62℃, 30s |
| *Yap1* | GCTGCAGCAGTTACAGATGG | TGCTCCAGTGTAGGCAACTG | 60℃, 30s |
| *Taz* | ACTGGCCAGAGATACTTCCTTAATC | AGGCTGATTCATCACCTTCCTG | 60℃, 30s |

*GAPDH*, glyceraldehyde phosphate dehydrogenase; *PCNA*, proliferating cell nuclear antigen; *Bcl2*, B cell leukemia/lymphoma 2; *SF1*, steroidogenic factor 1; *StAR*, steroidogenic acute regulatory protein; *P450scc*, cytochrome P450 cholesterol side chain cleavage; *3β-HSD1*: 3β-hydroxysteroid dehydrogenase-1; *CYP19,* cytochrome P450 family 19; *Nobox*, NOBOX oogenesis homeobox; *Figlα*, factor in the germline alpha; *BMP15,* bone morphogenetic protein 15; *GDF9*, growth differentiation factor 9; *Smad4,* SMAD family member 4; *Sohlh2*, spermatogenesis and oogenesis bHLH transcription factor 2; *Mst1*, macrophage stimulating 1; *Mst2*, macrophage stimulating 2; *Yap1*, yes-associated protein 1; *Taz,* transcriptional co-activator with PDZ-binding motif.
